# Effect of Pd-Sensitization on Poisonous Chlorine Gas Detection Ability of TiO_2_: Green Synthesis and Low-Temperature Operation

**DOI:** 10.3390/s22114200

**Published:** 2022-05-31

**Authors:** Satish Ekar, Umesh T. Nakate, Yogesh B. Khollam, Shoyebmohamad F. Shaikh, Rajaram S. Mane, Abu ul Hassan S. Rana, Marimuthu Palaniswami

**Affiliations:** 1 Department of Physics, Baboraoji Gholap College, Pune 411027, Maharashtra, India; ybkhollam@gmail.com; 2 Department of Polymer-Nano Science and Technology, Jeonbuk National University, 567 Baekje-daero, Deokjin-gu, Jeonju-si 54896, Jeollabuk-do, Korea; umesh.nakate@gmail.com; 3 Department of Chemistry, College of Science, King Saud University, Bld-5, Riyadh 11451, Saudi Arabia; sshaikh1@ksu.edu.sa; 4 Centre for Nano-Materials and Energy Devices, School of Physical Sciences, Swami Ramanand Teerth Marathwada University, Nanded 431606, Maharashtra, India; rajarammane70@srtmun.ac.in; 5 Department of Electrical and Electronic Engineering, The University of Melbourne, Parkville, VIC 3010, Australia; palani@unimelb.edu.au; 6 Department of Obstetrics and Gynaecology, The University of Melbourne, Parkville, VIC 3010, Australia

**Keywords:** bio-synthesis of TiO_2_, Cl_2_ sensor, Pd-sensitization, low-temperature, selectivity, gas sensor

## Abstract

Ganoderma lucidum mushroom-mediated green synthesis of nanocrystalline titanium dioxide (TiO_2_) is explored via a low-temperature (≤70 °C) wet chemical method. The role of Ganoderma lucidum mushroom extract in the reaction is to release the ganoderic acid molecules that tend to bind to the Ti^4+^ metal ions to form a titanium-ganoderic acid intermediate complex for obtaining TiO_2_ nanocrystallites (NCs), which is quite novel, considering the recent advances in fabricated gas sensing materials. The X-ray powder diffraction, field emission scanning electron microscopy, Raman spectroscopy, and Brunauer–Emmett–Teller measurements etc., are used to characterize the crystal structure, surface morphology, and surface area of as-synthesized TiO_2_ and Pd-TiO_2_ sensors, respectively. The chlorine (Cl_2_) gas sensing properties are investigated from a lower range of 5 ppm to a higher range of 400 ppm. In addition to excellent response–recovery time, good selectivity, constant repeatability, as well as chemical stability, the gas sensor efficiency of the as-synthesized Pd-TiO_2_ NC sensor is better (136% response at 150 °C operating temperature) than the TiO_2_ NC sensor (57% at 250 °C operating temperature) measured at 100 ppm (Cl_2_) gas concentration, suggesting that the green synthesized Pd-TiO_2_ sensor demonstrates efficient Cl_2_ gas sensing properties at low operating temperatures over pristine ones.

## 1. Introduction

Recently, controlling air pollutants and monitoring toxic gases have become increasingly important [1]. Concerns about the environment’s health need stringent regulations on the harmful gas emissions from industrial and social sectors. Chlorine (Cl_2_), one such toxic gas with a pungent odor, has been a great concern in the past few decades because it is extremely harmful to humans and the environment [2]. Cl_2_ molecules can combine with water in the lung mucosa to form hydrofluoric acid when contacted with Cl_2_ gas by breathing, causing serious respiratory difficulties. Exposure to high concentrations of Cl_2_ is fatal to the human body, irritates the eyes, causes skin infection, psychological disorders, and so on [3]. Cl_2_ is widely used in disinfectants, bleach, and raw materials to produce hydrochloric acid, and as part of various industrial processes, including tap water treatment, paper manufacturing, printing, and dyeing. When Cl_2_ is released into the environment without proper treatment, it can be negatively impacted [4]. Considering the growing concern about the hazardous effects of air pollutants, the fabrication and commercialization of low-cost toxic gas sensors is a prime mandate for environmental protection [5].

Over the decades, semiconducting wide band-gap metal oxides have been envisaged in biomedical, water purification, solar cell, chemical and biological sensor applications, and so on [6]. Semiconducting metal oxides such as ZnO [7], SnO_2_ [8], WO_3_ [9], Fe_2_O_3_ [10], Bi_2_O_3_ [11], and so on, have widely been employed in different gas sensor studies. Among them, TiO_2_ has gained attention among solid-state sensors due to its low-cost, eco-friendly nature, non-toxic nature, different crystal structures, such as rutile, anatase, and brookite with different chemical, electrical, structural, and optical properties, availability in different dimensions, compatibility, and simplicity of fabrication technology [12,13]. It is an *n*-type semiconducting wide-band gap material with promising applications in gas detection, solar cells, self-cleaning glasses, water purification, and food product industries. It adduces potential biological activities such as anti-fungal, anti-cancer, and anti-microbial. Furthermore, the gas sensing properties of these metal oxide sensors in terms of fast response, low-operating temperature, and good selectivity can be improved by decorating them with nanoparticles of noble metals such as gold (Au), platinum (Pt), and palladium (Pd) [14,15,16]. Several methods are being established for obtaining TiO_2_ nanostructures, such as chemical bath deposition, spray pyrolysis, anodization, sol-gel, hydrothermal, and so on. The bio-inspired metal oxide nanoparticles synthesized using green chemistry demonstrates several advantages over the chemical and physical approaches as this process is; simple (one-pot), low-temperature (70 °C) operating, cost-effective, and environmentally friendly. Furthermore, the abundance and accessibility of biological precursors facilitate large-scale technology development. Green synthesis eliminates the need for additional capping or stabilizing agents, lowering fabrication cost and simplifying the synthetic process. Although green chemistry has several advantages over synthetic chemical approaches, some major concerns must be addressed before these methodologies can be commercialized on a large scale. The main concerns are the possibility of ecological imbalance in natural bioresources due to overuse; differences in active biomolecule/phytochemical concentrations due to seasonal or climate changes, which affect bioactivity or synthesis procedures; and the fact that several nanoparticles (NPs) cannot be prepared using the biosynthesis method due to the requirement of strong reducing agents [17]. Present work is novel in terms of both the green synthesis of TiO_2_ nanocrystallites (NCs) and their low-temperature Cl_2_ gas sensor application. Efforts have also been taken to enhance the selectivity and gas sensing properties of TiO_2_ NCs through Pd-sensitization. Wu et al., prepared Pd-doped mesoporous WO_3_ through a hard-templating process with potential hydrogen gas sensing capabilities, such as fast response time, excellent reproducibility, and quick recovery time [18]. The Pd-modified SnO_2_ NCs sensor showed improved gas-sensing characteristics, allowing carbon monoxide detection and other extremely low concentration reducing gases such as ethanol and acetone [19]. Suhail et al., obtained a Pd-doped ZnO hydrogen gas sensor; wherein, the doped ZnO film response time of 2–3 s was significantly faster than the traditional ZnO sensor [20]. Pandey et al., investigated the significance of Pd and reduced graphene oxide layers on the gas sensor response of toluene, CO, and ethanol for hydrogen selectivity [21]. At room-temperature, Zhao et al. produced the CdSnO_3_ gas sensor with improved sensing properties to 1–10 ppm of chlorine gas; the value of gas response was 1338.9 to 5 ppm [22]. Hieu et al., discovered that SnO_2_ nanowires are more sensitive to Cl_2_ gas than ZnO and WO_3_ nanowires. The SnO_2_ nanowire sensor that responded to 50 ppb Cl_2_ at 50 °C was about 57 [23]. According to Stadler et al., ZnO nanoparticles exhibited a reasonable response of 1278 percent to 200 ppm Cl_2_ at 200 °C, with fast response and recovery time periods [24].

In the present work, the TiO_2_ sensor is successfully synthesized via a low-temperature (≤70 °C) bio-inspired wet chemical synthesis method in the presence of Ganoderma lucidum mushroom for Cl_2_ gas sensing application. The role of Ganoderma lucidum mushroom extract in the reaction is to release the ganoderic acid molecules, which tend to bind the Ti^4+^ metal ions to form a titanium–ganoderic acid intermediate complex for obtaining TiO_2_ NCs. The Pd-sensitization is carried out on the TiO_2_ sensor surface by impregnating the Pd-TiO_2_ NC sensor. Both TiO_2_ and Pd-TiO_2_ NC sensors are characterized for their structure and morphology using standard material characterization tools. The gas sensing behaviors of TiO_2_ and Pd-TiO_2_ NC sensors are investigated for various gases such as ammonia (NH_3_), hydrogen sulfide (H_2_S), carbon dioxide (CO_2_), nitrogen dioxide (NO_2_), and chlorine (Cl_2_), out of which, Cl_2_ has revealed an optimum performance, and therefore, the Cl_2_ sensing properties are performed as a function of operating temperature and concentration with error limit. The transient responses for both sensors are also studied to find the respective response and recovery periods. The repeatability and stability tests of both sensor materials are recorded and reported.

## 2. Materials and Methods

### 2.1. Materials

The analytical grade titanium (IV) isopropoxide (97%, TTIP), palladium (II) chloride (99%, PdCl_2_), polyvinyl alcohol (PVA, [-CH_2_CHOH-]n), ethyl alcohol(96%, CH_3_CH_2_OH) are obtained from Sigma Aldrich and utilized as received, without further purification.

### 2.2. An Extract Preparation

The medicinal Ganoderma lucidum mushroom collected from the forest of Tamhini of Pune District, Maharashtra, India, was washed with distilled water (Milli-Q water; 18.2 MΩ cm) a few times to remove dust and any other parasites that could be present. It was then dried under shade at room-temperature (27 °C) for about fifteen days under dust-free conditions. The mushroom was then divided into fine pieces, grounded, and sieved to get a fine powder. For 15 min, 1.0 gm of fine mushroom powder was boiled in 100 mL of distilled water at 85 °C. The resulting solution was filtered through Whatman paper no. 1 of 42 pore size and stored as a stock solution at 4 °C before use.

### 2.3. Synthesis of TiO_2_ NC Powder

**Scheme 1:**
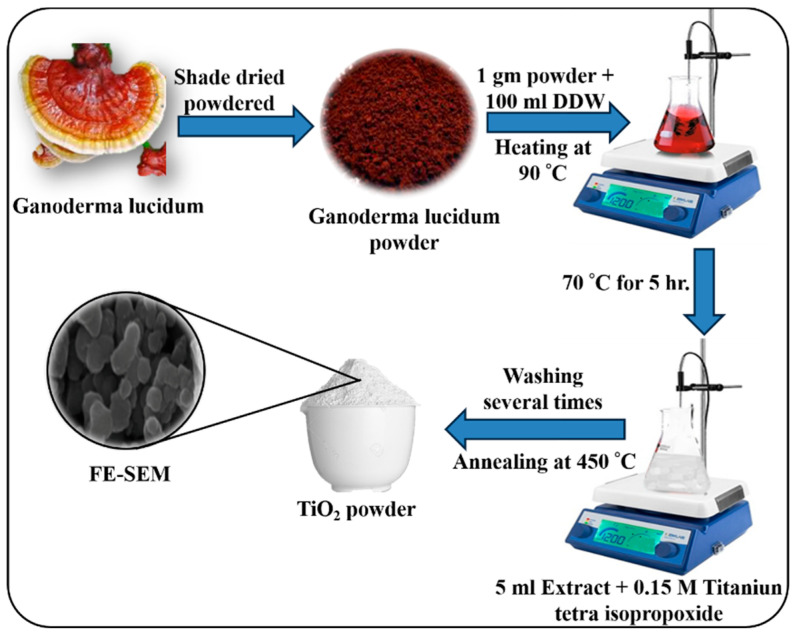
Schematic presenting a process for obtaining bio-inspired TiO_2_ NCs.

To prepare Ti- precursor, the 0.5 M titanium (IV) isopropoxide was added into 50 mL of ethyl alcohol and distilled water (1:1 proportion). The mushroom extract solution (5 mL) was added into the Ti-precursor solution, and the resulting mixture was stirred for 5 h and maintained at 70 °C by using a hot plate magnetic stirrer. The solution was allowed to cool down to room temperature (27 °C) after vigorous stirring, and the supernatants were removed, leaving a brown-yellow precipitate as a residue. The precipitate was washed several times with ethyl alcohol and distilled water. The color of the precipitate was brown-yellow due to the formation of the titanium-ganoderic acid intermediate compound. The synthesized precipitate was dried at 50 °C overnight. Finally, this fried precipitate was air-annealed at 450 °C for 2 h to improve the crystallinity of white TiO_2_ powder before being kept in an airtight container for later usage (Scheme 1). The solid-state sensor film was prepared by dissolving 1.0 g of polyvinyl alcohol (PVA) as a binder in 10 mL of distilled water followed heating the solution to 90 °C while constantly stirring until a transparent viscous gel. The TiO_2_ NCs powder was mixed with a few drops of viscous PVA solution in a pestle and mortar. The excess water and binder were vaporized by coating the mixture on the conventional soda-lime glass as a substrate and drying it at 200 °C for 1h. Half of the obtained TiO_2_ film sample was considered pristine TiO_2,_ whereas the remaining half was used for Pd-sensitization as Pd-sensitized TiO_2_.

### 2.4. Growth Mechanism

TiO_2_ NCs were synthesized through the hydrolysis of titanium (IV) isopropoxide in the presence of ganoderic acid at 70 °C as follows. The Ti(OC_3_H_7_)_4_ reacts with water. The amount of water determines the degree of hydrolysis (i.e., solubility and ionic products), which is high at the degree of saturation where both are nearly the same, and the type of initial species formed, such as ganoderic acid molecules for binding metal ions, thus influencing the condensation reaction that involves the polymerization of hydrolyzed titanium alkoxides in alcoholic solution to form Ti^+4^ ions in the solution. Thus, the Ti^+4^ ions bound to ganoderic acid legends to form the titanium–ganoderic acid intermediate complex. The titanium–ganoderic acid complex molecules most likely produce a steric hindrance at specific places via hydrogen bonding, helping to control both the particle size and the porous nature of the aggregates. Subsequently, the as-developed titanium-ganoderic acid complex powder was annealed at 450 °C to obtain a very fine-sized TiO_2_ NCs powder.

### 2.5. Pd-Sensitized TiO_2_ NCs

In the present work, Pd-sensitized TiO_2_ film was fabricated as follows. The 0.01M PdCl_2_ in an aqueous solution was used as metal salts for the sensitization of TiO_2_ film. The TiO_2_ film prepared using the doctor blade method was dipped into a 0.01M aqueous solution of the respective salt for 5 s, and then dried under an IR lamp. Ten such cycles of dipping were carried out for each film. The sensitized TiO_2_ film was then heated at 200 °C for 1 h to remove the chloride from the deposited sensor film.

### 2.6. Characterization Techniques

The field emission scanning electron microscope (FE-SEM, Nova-SEM 200-FEI) was used to confirm the surface morphology of TiO_2,_ and Pd-TiO_2_ sensor film surfaces A FEI TECNAI G2 20 STWIN equipment was used to capture the High-Resolution Transmission Electron Microscopy (HR-TEM) image and electron mapping configuration. The crystal structure was elucidated using an X-ray diffraction (XRD) spectrum (XRD-6000, Shimadzu X-ray diffractometer) and a Cu-K radiation tube. The elemental contents were identified by using an X-ray photoelectron spectroscopy profile obtained on a PHI 5000 Versa Probe (XPS, Ulvac-PHI). Additionally, the Belsorp II, BET, and Japan Inc. equipment measured the surface area and pore-size distribution.

### 2.7. Gas Sensor Setup

The gas sensing operation was performed in a stainless-steel chamber with 250 mL volume capacity. To adjust the constant temperature, a PID-controlled heater was installed at the base of the cylindrical chamber. To mitigate against changes in operating temperature, a dimmer stat was employed as a stable voltage supply. After adding the target gases to the stainless-steel chamber, a Keithley 6514 programmable computer-connected electrometer was utilized to identify the sensor film resistance. Thin films with an area of 1.5 × 1.5 cm^2^ were made using a doctor-blade technique on a non-conducting glass substrate in presence of PVA as the binder material which was air-annealed for sensor activity measurement (200 °C, 1 h). The commercial silver paste was used to draw the electrical contacts. The following equation was used to calculate the gas sensor response:

(1) $$S\left(\%\right)=\frac{\left|R_{a}-R_{g}\right|}{R_{a}}\times 100$$

where *R_a_* represents the stabilized resistance of ambient air, *R_g_* represents the stabilized resistance of the target gas.

## 3. Results and Discussion

### 3.1. Surface Morphology Appearance

**Figure 1:**
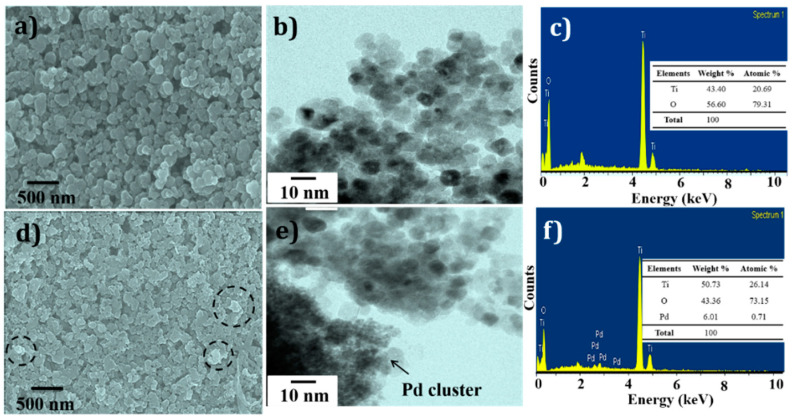
FE-SEM, HR-TEM; elemental analysis of (**a**–**c**), TiO_2_, and (**d**–**f**); Pd-TiO_2_ sensor surfaces.

The FE-SEM micrographs of the as-obtained TiO_2_ and Pd-TiO_2_ NC film sensors at two different magnifications are shown in Figure 1a,d. The FE-SEM image of the TiO_2_ film sensor demonstrates the formation of small-sized spherical nanocrystallites with an agglomerated-type architecture. The Pd nanoparticles are uniformly dispersed over the TiO_2_ film sensor surface in the form of soft agglomerations. Pd nanoparticles show a spillover effect on the surface of the TiO_2_ film, which is discussed in the below section. The crystal structure and phase analysis of TiO_2_ and Pd-TiO_2_ NC sensors characterized by using HR-TEM and HR-TEM techniques are shown in Figure 1b,e. Figure 1b presents the HR-TEM image of TiO_2_, where the particle size is about 8.0 nm, which was an exact estimation of the XRD (101) peak (i.e., 7.2 nm) using Scherrer relation. Figure 1e provides the HR-TEM image of the Pd-TiO_2_ sensor where the presence of Pd NCs in the TiO_2_ matrix is evident. Pd in the form of the cluster along with TiO_2_ NCs is noticed. The average particle size of the Pd nanocluster (~2 nm) is estimated from the HR-TEM images of Pd-TiO_2_ NCs (Figure 1e). The surface elemental stoichiometry measured from EDX profile reveals 20.69 and 79.31 percent for the atomic proportions of Ti and O present in the TiO_2_ film sensor, whereas the mapping proportions for Ti, O, and Pd in Pd-TiO_2_ are respectively 26.14, 73.15, and 0.71 (Figure 1c,f).

### 3.2. Structural Elucidation

**Figure 2:**
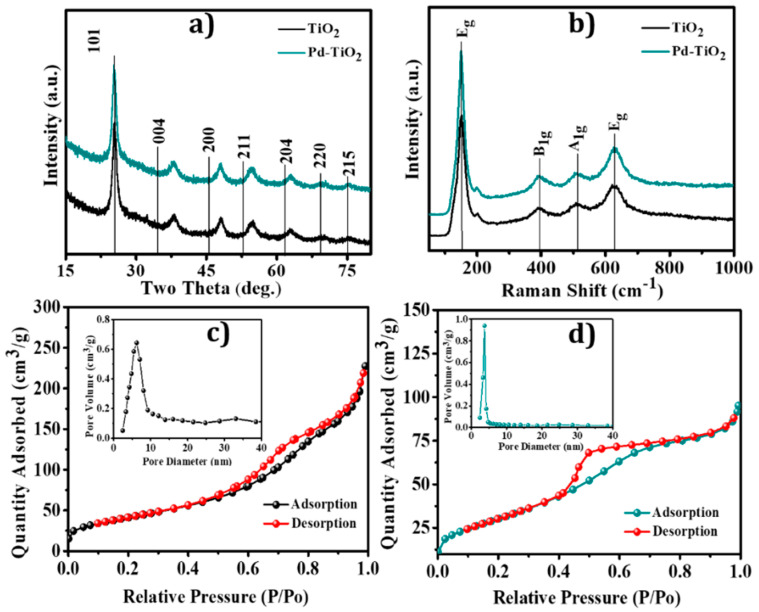
(**a**) XRD patterns, (**b**) Raman spectrums of TiO_2_ and Pd-TiO_2_ sensors, and (**c**,**d**) respective BET adsorption–desorption isotherms with pore-size distribution measurements (inset).

The XRD patterns of TiO_2_ and Pd-TiO_2_ film sensors are shown in Figure 2a. The appearance of the diffraction peaks in the XRD patterns confirms the formation of pyramidal crystal structure with anatase TiO_2_ phase (JCPDS card No. 21-1272) [25]; however, due to the infinitesimal amount, there is no evolution of diffraction peaks in the XRD pattern for Pd. The sharp diffraction peaks indicate good crystallinity and involvement of no impurity peaks [26]. The metal oxide gas sensor properties can be influenced by structural parameters such as crystallite size, texture coefficient (TC), and dislocation densities; hence, there is a need to study these parameters of given sensor materials [27,28,29]. The estimated structural parameters of the as-obtained TiO_2_ film sensor are tabulated in Table 1. The dislocation densities calculated for each diffraction peak are also given in Table 1. The TC values corresponding to every diffraction peak were estimated. If the TC value is greater than one, sugegsting the preferred orientation. From Table 1, the maximum TC value of 1.22 is associated with the (101) plane, which is the preferred growth direction of the present TiO_2_ film sensor. The negative value of micro-strain observed in Table 1 infers the existence of compressive type strain. The dislocation densities corresponding to each diffraction peak are mentioned in Table 1.

Raman spectroscopy is a powerful technique to detect molecules in TiO_2_ film sensor obtained from the Ganoderma lucidum mushroom. The significant Raman shift peaks at E_g_ (144), E_g_ (205), B_1g_ (400), B_1g_/A_1g_ (519), and E_g_ (635) cm^−1^, as shown in Figure 2b, are attributed to the anatase crystal phase of TiO_2_ [30]. In the Raman spectrum, no additional peaks, except distinctive vibrations, are found, thus confirming the formation of the pure phase of Pd-TiO_2_. The nitrogen adsorption and desorption isotherms obtained during the BET surface area measurement for pristine TiO_2_ and Pd-TiO_2_ films are shown in Figure 2c,d. The specific surface areas for TiO_2_ and Pd-TiO_2_ NC film sensors are respectively 161.5 m^2^/g and 170.9 m^2^/g. It seems that Pd incorporation decreases the particle size of TiO_2_, which, in turn, enhances the specific surface area [31]. The insets of Figure 2c,d present the pore-size distribution curves for the pristine TiO_2_ and Pd-TiO_2_ NCs sensors. The pore-size distribution is narrow, nearly nano-sized pores, resulting in an average pore volume of 0.15 cm^3^/g for pristine TiO_2_ and 0.17 cm^3^/g for Pd-TiO_2_ NCs. The average pore diameter of the pristine TiO_2_ and Pd-TiO_2_ NCs are found to be 7.13 and 4.25 nm, respectively. The higher surface area of pristine TiO_2_ and Pd-TiO_2_ NCs and their porous nature may provide access to active sites and a surface for adsorption of target gas molecules to enhance the gas sensing activity.

### 3.3. Elemental Analysis

**Figure 3:**
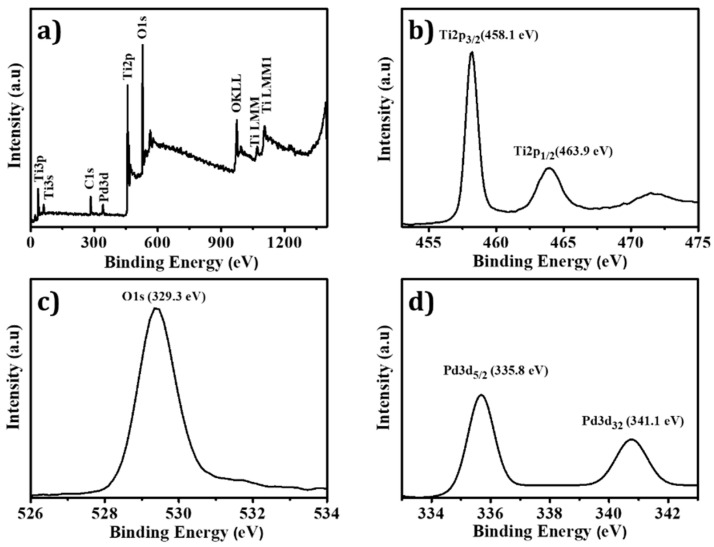
XPS analysis of Pd-TiO_2_ film sensor; (**a**) survey spectrum, and (**b**–**d**) high-resolution Ti2p, O1s, and Pd 3d spectrums.

The surface states of Pd-TiO_2_ NCs were investigated by using X-ray photoelectron spectroscopy analysis. The survey XPS spectrum of the Pd-TiO_2_ film sensor is shown in Figure 3a, confirming the presence of Pd, Ti, and O elements. The high-resolution XPS spectra for Ti2p, O1s, and Pd 3d are shown in Figure 3b–d.

The Ti2p_3/2_ peak has a binding energy of 463.9 eV, whereas the low-intensity Ti2p_1/2_ peak has a binding energy of 458.1 eV, thus indicating the presence of a Ti^4+^ oxidation state [32]. The O1s spectrum of the pristine TiO_2_ film sensor noted, as seen in Figure 3c at 529.3 eV, is of TiO_2_ which is ascribed to the surface oxygen atoms [33]. The Pd3d spectrum, as shown in Figure 3d, adduces two peaks: one at 341.1 eV corresponding to Pd3d_3/2_, and the other at 335.8 eV for Pd3d_5/2_. The appearance of Pd^2+^ peak may be due to the partial oxidation of the Pd surface since the Pd particles are very minute in the Pd-TiO_2_ film sensor [34].

### 3.4. Gas Sensing Properties

**Figure 4:**
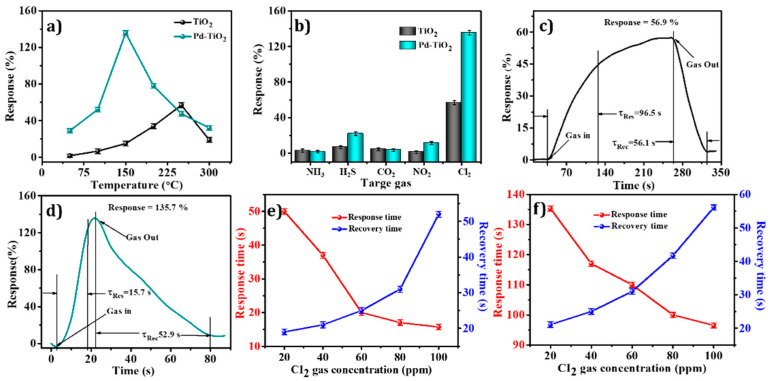
(**a**) Gas response (at different operating temperatures); (**b**) selectivity of sensors for various gases; (**c**,**d**) response and recovery measurements at 100 ppm of Cl_2_ gas; and (**e**,**f**) response vs. recovery plots for Cl_2_ gas at different concentrations (from 20 to 100 ppm) of TiO_2_ and Pd-TiO_2_.

The gas sensing properties of the TiO_2_ and Pd-TiO_2_ film sensors were investigated. Metal oxide-based gas sensors usually work at higher operating temperatures (i.e., ≥150 °C). The adsorption/desorption of target gas molecules varies with the operating temperature. As a result, optimizing the current sensor’s operating temperature is critical. These TiO_2_ and Pd-TiO_2_ sensors were studied for Cl_2_ (100 ppm) at various operating temperatures (Figure 4a). It was observed that the TiO_2_ film sensor reveals the highest Cl_2_ response of 57%, at a 250 °C operating temperature. In contrast, the Pd-TiO_2_ film sensor demonstrates the highest Cl_2_ response at 136%, at a relatively low operating temperature (i.e., 150 °C). The involvement of oxygen vacancies, which act as defects, is responsible for an *n*-type semiconducting behavior of TiO_2_; hence, electrons are the majority charge carriers in the conduction band. The basis for sensor working is the change of its resistance on the exposure of the target gas. The resistance of the TiO_2_ NCs film sensor is increased after Cl_2_ gas exposure. A typical Cl_2_ gas sensing mechanism has been explained for *n*-type metal oxide-based gas sensor by Navale et al. [24]. The presence of the Pd catalyst on the TiO_2_ surface is responsible for the higher Cl_2_ gas response at the relatively low optimal operating temperature [15]. Pd (being noble metal) has better dissociation catalytic ability.

The presence of Pd catalyst on the sensor surface provides an active site and surface to enhance the catalytic chemical sensing reaction. The electrical and chemical effects of the Pd catalyst help to improve the gas sensing performance [35]. As well as Cl_2_, these sensors are further employed to check their responses to gases such as NH_3_, H_2_S, CO_2_, and NO_2_ (Figure 4b). Both sensors feature good selectivity towards Cl_2_ gas, whereas the Pd-TiO_2_ NCs film sensor reveals higher selectivity over the TiO_2_ film sensor. The transient Cl_2_ gas responses for TiO_2_ and Pd-TiO_2_ film sensors are provided in Figure 4c,d. As the Cl_2_ gas is injected into the testing system, the target gas molecules diffuse through air and get adsorbed onto the sensor surface for catalytic sensing reaction over time. This sensor shows the response in increasing order as time progresses until saturation or equilibrium level for a given target gas concentration is achieved.

At the saturation level, the sensor shows a constant response. After the gas testing system is opened to an outer atmosphere, the target gas molecules start desorbing, and the corresponding sensor response also decreases. The time taken by the sensor to reach 90% of the change in response or resistance value for a given concentration of target gas is nothing but the response time. Similarly, recovery time can also be recorded. The recorded response time values for TiO_2_ and Pd-TiO_2_ sensors are respectively 96.5 s and 15.7 s. The recovery time values for TiO_2_ and Pd-TiO_2_ sensors are 56.1 s and 52.9 s, respectively, which are approximately the same. The fast response of the Pd-TiO_2_ NC film sensor is attributed to the fast Cl_2_ gas sensing catalytic reaction on the sensor surface in the presence of Pd. The repeatability and transient gas response studies for increasing the target gas concentration of TiO_2_ and Pd-TiO_2_ sensors are given in Figure 4e,f. In Figure 4f, the response time decreases from 135 to 96.5 s for the Pd-TiO_2_ NC film sensor, whereas recovery time increases from 21 to 56 s with an increase of Cl_2_ gas concentration from 20 to 100 ppm. The decrease in response time is attributed to the abundance of vacant sites on the film surface for gas adsorption. At the same time, the increase in recovery time is assigned to the heavier nature of Cl_2_ gas. Moreover, this may be due to the reaction products that are not leaving the interface immediately after the reaction, thus resulting in a decrease in desorption rate [36].

### 3.5. Effect of Cl_2_ Gas Concentration on Sensor Response

**Figure 5:**
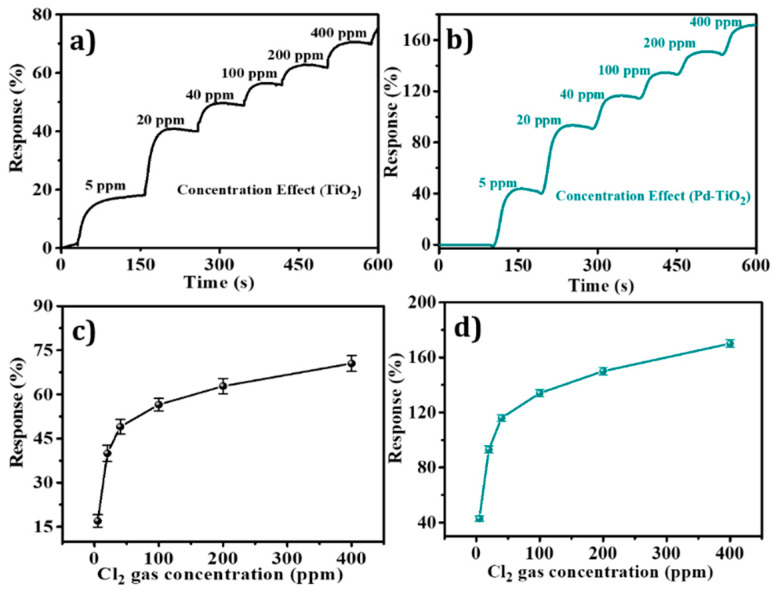
(**a**,**b**) Dynamic response–recovery characteristic curves at different Cl_2_ gas concentrations (from 5 to 400 ppm) and (**c**,**d**) exponential relationship (between gas response and gas concentration) of TiO_2_ and Pd-TiO_2_ NC film sensors.

Figure 5a,b depict the dynamic gas responses of the TiO_2_ and Pd-TiO_2_NCs’ film sensors vs. time towards 5–400 ppm concentrations of Cl_2_ gas. The response values of the TiO_2_ sensor toward 5, 20, 40, 100, 200, and 400 ppm concentrations of Cl_2_ gas at 250 °C are found to be 17.14, 40.8, 49.6, 56.9, 62.7, 68.2, and 70.4%, respectively. It is observed that, as the concentration of Cl_2_ increases, the TiO_2_ film sensor response also increases; this is because higher Cl_2_ gas concentration covers the higher surface of the TiO_2_ film sensor, eventually increasing the interaction between Cl_2_ gas molecules and sensor surface. On the other hand, lower Cl_2_ gas concentration mitigates the interaction between Cl_2_ gas molecules and the sensor surface; a lower response is obtained due to the lower surface coverage [37]. Moreover, from Figure 5b, the Pd-TiO_2_ NC film sensor can detect as low as 5 ppm Cl_2_ gas with a phenomenal response of 40.3%. Both the sensors demonstrate a lower gas response for lower Cl_2_ gas concentration. With an increase in Cl_2_ concentration, corresponding, responses are also increased (168.5% at 400 ppm). The response to maximum Cl_2_ gas concentration is restricted by the available area of the sensor surface. As the sensor surface area is limited, the sensor gets saturated, indicating a saturated response for higher gas concentrations [38].

As shown in Figure 5c,d, the exponential relationship between gas response and gas concentration—tested three times—reveals good reproducibility for both sensors. At lower concentrations, the Cl_2_ gas molecules cover a smaller portion of the surface of sensitized sensor films, resulting in fewer surface interactions and, as a result, a smaller response value is noted. Furthermore, a higher concentration of Cl_2_ gas covers more sensor surface area, resulting in a higher response value due to greater surface contacts [39]. The Cl_2_ gas sensing performance of the Pd-TiO_2_ NCs sensor was compared with previously published data (shown in Table 2).

### 3.6. Reproducibility and Stability Studies

**Figure 6:**
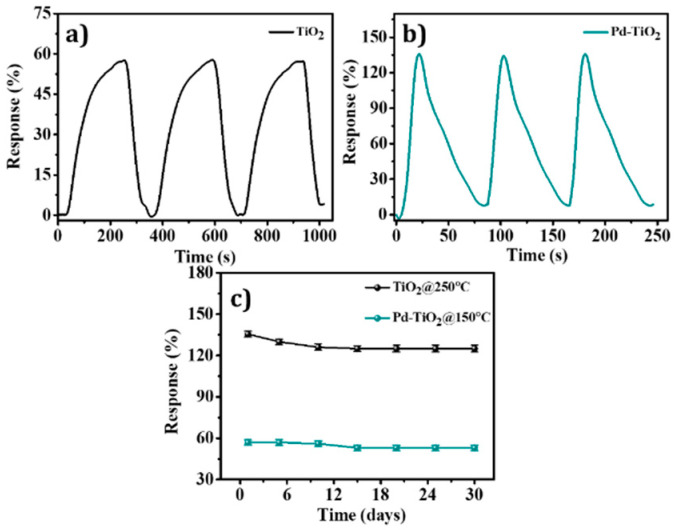
(**a**,**b**) Three response curves, and (**c**) long-term stability curve at 100 ppm Cl_2_ gas concentration of TiO_2_ and Pd-TiO_2_ film sensors.

The reliability and quality studies of the film sensors are represented by two important parameters; reproducibility and stability. The capability of sensor material to reproduce the same experimental results measured for another synthesis batch under similar working environmental conditions is reproducibility. To study the reproducibility of the TiO_2_ film sensor, three sensor scans were operated to 100 ppm of Cl_2_ gas in the same working environment (Figure 6a). The reproducibility of a sensor is one of the measures of its product quality. To investigate the reproducibility of the Pd-TiO_2_ NCs; film, response experiments were repeated three times towards a 100 ppm of Cl_2_. The results in Figure 6b demonstrate that upon repeated exposure to 100 ppm Cl_2_ gas, the response of the Pd-TiO_2_ NCs film sensor is almost constant, which confirms the excellent repeatability of the Pd-sensitized sensor material [40]. Response stability is one of the most important parameters of metal oxide-based gas sensors; therefore, the stability studies of both TiO_2_ and Pd-TiO_2_ NCs film sensors were studied towards the fixed 100 ppm concentration of Cl_2_ gas for thirty days at five days (Figure 6c). Both the sensors retain the responses significantly for thirty days; however, the Pd-TiO_2_ film sensor has confirmed the highest stability response (92.0%) to 100 ppm Cl_2_ gas concentration at 150 °C, thus signifying its excellent chemical and environmental stability.

### 3.7. Gas Sensor Working Mechanism

**Scheme 2:**
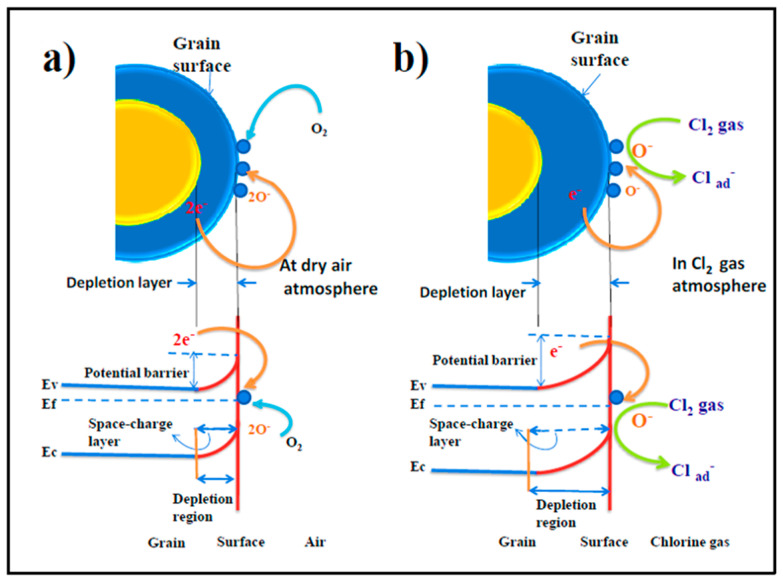
(**a**,**b**) Proposed sensing-based reaction mechanism for the Pd-TiO_2_ NC film sensor in air and Cl_2_ gas atmospheres.

The most widely prevalent gas sensor working mechanism for *n*-type metal oxide films is the sensor materials’ noteworthy resistance change, which is caused by the adsorption/desorption process of the gas molecules on the sensing materials’ surface during exposure to various gas environments [41]. Based on the energy band diagram, as given in Scheme 2, a gas sensing mechanism of the Pd-TiO_2_ film sensor in the presence of air atmosphere and Cl_2_ gas has been proposed. The majority charge carrier in the *n*-type Pd-TiO_2_ semiconductor is electrons. Under ambient air conditions, oxygen molecules are adsorbed on the Pd-TiO_2_ film’s exposed surface by capturing electrons from the TiO_2_ valance band. The process creates a surface depletion layer via adsorbed oxygen species (O^−^).

The resistance of the sensor films changes when oxygen is absorbed on the sensor surface. Generally, the adsorbed oxygen ion species depend on the sensor film temperature, with O^2−^ below 100 °C, O^−^ in between 100–300 °C, and O^2−^ above 300 °C [42]. O^−^ is dominant in the present study because of the approximate Pd-TiO_2_ film’s working temperature of 150 °C. From Scheme 2, it is seen that the potential barrier has a direct relationship with the oxygen ions on the film surface. The increase in potential barrier height eventually increases the film resistance. When the sensors are exposed to oxidizing gas, such as Cl_2_, Cl_2_ gas molecules react with oxygen ions to form ClO^−^ species, and conduction electrons (drawn from Pd-TiO_2_ film) confirm Cl_ad_^−^. This decreases the film’s carrier concentration, thus increasing the depletion width and film resistance. The proposed reaction is as follows:

(2) $$\frac{1}{2}{\mathrm{Cl}}_{2}+{\;e}^{-}\to {\mathrm{Cl}}_{\mathrm{ad}}{}^{-}$$

(3) $$\frac{1}{2}{\mathrm{Cl}}_{2}+V_{O\;}+e^{-}\to {\mathrm{ClO}}^{-}$$

where subscripts ad^−^ and O^−^ denotes species adsorbed on the surface of the film sensor and species occupy lattice oxygen sites, respectively, and Vo is oxygen vacancy [27,43]. The proposed gas sensing mechanism of the Pd-TiO_2_ NCs’ film sensor for the Cl_2_ gas atmosphere is shown in Scheme 2b. Noble metals such as Au, Pt, Ag, and Pd are frequently used as sensitizers in sensing materials to improve their gas sensing properties [44]. The enhanced gas sensing properties of nobel metal doped sensing materials are due to two effects; electronic sensitization and chemical sensitization [45]. To begin, in the case of the electron sensitization effect, free electron transfer between Pd and TiO_2_ occurs due to their different work functions, resulting in the formation of a Schottky barrier and extra electron depleted layers near the contact interfaces. When the Cl_2_ gas molecules come into contact with the sensing material, the extra electron depleted layer causes an increasing number of Cl_2_ molecules to be adsorbed on the sensor surface, thus increasing the gas sensor response. Second, the active Pd nanoparticles accelerate the reaction by providing more active sites for Cl_2_ gas molecules to interact with, which may be one of the factors responsible for improved sensor performance.

## 4. Conclusions

In summary, the nanocrystalline TiO_2_ film sensor has been synthesized by a low-temperature (≤70 °C) bio-inspired wet chemical method in Ganoderma lucidum mushroom sensitized with Pd as Pd-TiO_2_ sensor by a simple dip-coating method. Both the sensors endow a good selectivity for Cl_2_ gas. The slow response/recovery time and high operating temperature are also major drawbacks of Cl_2_-based gas sensors. To overcome this disadvantage, Pd doping in TiO_2_ produces a synergetic effect that improves response/recovery time and lowers operating temperature. The TiO_2_ sensor shows the highest Cl_2_ gas response of 57% for 100 ppm at 250 °C operating temperature. The Pd-TiO_2_ film sensor exhibits the highest Cl_2_ gas response of 136% at the relatively lower operating temperature of 150 °C. The response/recovery time for the TiO_2_ sensor is 97 s/56 s, whereas it is just 16 s/52 s for the Pd-TiO_2_ film sensor. At a concentration of 5 ppm, Cl_2_ detection is smaller, whereas, at a concentration of 400 ppm, Cl_2_ detection is higher. The present sensors reveal a good response–recovery properties, selectivity, remarkable repeatability, and stability for Cl_2_ sensing; therefore, bio-inspired wet chemical synthesis of TiO_2_ in the presence of Ganoderma lucidum mushroom can be a potential candidate, over other expensive and rare-earth materials, for obtaining TiO_2_ and Pd-TiO_2_ film sensors which would be an easy way to increase the sensitivity of Cl_2_ for producing scalable commercial gas sensors.

## Figures and Tables

**Table 1:** Structural parameters of TiO_2_ NCs.

Plane (hkl)	I(hkl)/I0(hkl)	Texture Coefficient	Average Crystal Size D (nm) $D=\frac{K.\lambda }{\beta .\cos\theta }$	Micro-Strain ε × 10^−3^	Dislocation Density Lines/m^2^ δ × 10^15^ δ = 1/D_2_
(101)	11.61	1.12	8.9	−7.19	12.60
(004)	8.9	0.84	6.8	−6.69	21.60
(105)	10.2	0.99	7.6	−7.35	17.31
(204)	10.0	0.97	5.4	−11.93	34.29

**Table 2:** Comparison of responses of different material-based Cl_2_ gas sensors found in the literature. (C = Concentration (ppm), S = Response, T = Temperature (°C), RT = Room temperature).

Material	Synthesis Method	Gas	S	C	T (°C)	Ref.
CdSnO_3_	Hydrothermal	Cl_2_	1338.9 *	5 ppm	RT	[22]
SnO_2_	Chemical vapor deposition	Cl_2_	57 *	50 ppb	50 °C	[23]
ZnO	Rapid solid-state	Cl_2_	1278%	200 ppm	200 °C	[24]
**Pd-TiO_2_**	**Wet chemical**	**Cl_2_**	**136%**	**100 ppm**	**150 °C**	**This Work**

* The Response value calculated using formula S = R_a_/R_g_.

## Data Availability

Not applicable.
